# Immunization of Newborn Mice Accelerates the Architectural Maturation of Lymph Nodes, But AID-Dependent IgG Responses Are Still Delayed Compared to the Adult

**DOI:** 10.3389/fimmu.2017.00013

**Published:** 2017-01-19

**Authors:** Rosario Munguía-Fuentes, Juan Carlos Yam-Puc, Aarón Silva-Sánchez, Edith Marcial-Juárez, Isis Amara Gallegos-Hernández, Juana Calderón-Amador, Troy D. Randall, Leopoldo Flores-Romo

**Affiliations:** ^1^Department of Cell Biology, Center for Advanced Research, The National Polytechnic Institute, Cinvestav-IPN, Mexico City, Mexico; ^2^Department of Medicine, Division of Clinical Immunology and Rheumatology, University of Alabama at Birmingham, Birmingham, AL, USA

**Keywords:** neonates, immunization at birth, FDCs, FRCs, germinal centers, class-switched antibodies

## Abstract

Lymph nodes (LNs) have evolved to maximize antigen (Ag) collection and presentation as well as lymphocyte proliferation and differentiation—processes that are spatially regulated by stromal cell subsets, including fibroblastic reticular cells (FRCs) and follicular dendritic cells (FDCs). Here, we showed that naïve neonatal mice have poorly organized LNs with few B and T cells and undetectable FDCs, whereas adult LNs have numerous B cells and large FDC networks. Interestingly, immunization on the day of birth accelerated B cell accumulation and T cell recruitment into follicles as well as FDC maturation and FRC organization in neonatal LNs. However, compared to adults, the formation of germinal centers was both delayed and reduced following immunization of neonatal mice. Although immunized neonates poorly expressed activation-induced cytidine deaminase (AID), they were able to produce Ag-specific IgGs, but with lower titers than adults. Interestingly, the Ag-specific IgM response in neonates was similar to that in adults. These results suggest that despite an accelerated structural maturation of LNs in neonates following vaccination, the B cell response is still delayed and reduced in its ability to isotype switch most likely due to poor AID expression. Of note, naïve pups born to Ag-immunized mothers had high titers of Ag-specific IgGs from day 0 (at birth). These transferred antibodies confirm a mother-derived coverage to neonates for Ags to which mothers (and most likely neonates) are exposed, thus protecting the neonates while they produce their own antibodies. Finally, the type of Ag used in this study and the results obtained also indicate that T cell help would be operating at this stage of life. Thus, neonatal immune system might not be intrinsically immature but rather evolutionary adapted to cope with Ags at birth.

## Introduction

Neonates are often thought to be immunologically immature (1, 2), in part due to anti-inflammatory dendritic cells (3, 4) and immature lymphocytes (4–6). However, secondary lymphoid organs (SLOs), which bring antigen (Ag)-presenting cells into contact with their cognate lymphocytes and provide niches for the differentiation of lymphocytes, are also incompletely developed in neonates. The architecture of SLOs is supported by non-hematopoietic stromal cells (7), including fibroblastic reticular cells (FRCs) and follicular dendritic cells (FDC) (7, 8). Nevertheless, it is not clear how these cells influence the architecture of neonatal SLOs or whether early exposure to Ag affects the maturation of these cells (7, 8).

In adults, the FRC network extends from the subcapsular sinus (SCS) of the lymph node (LN) through the entire T-cell zone and is contiguous with high endothelial venules (HEVs) (9). FRCs maintain lymphocyte homeostasis *via* the production of IL-7 (10–12) and direct leukocyte traffic *via* chemokine secretion (13–15). FRCs also form a conduit system through which the LNs can collect small molecules (12). Importantly, FRCs provide strength and flexibility to LNs and allow them to be restructured following inflammation, thereby providing space for the influx or proliferation of lymphocytes following antigenic exposure (16). In contrast to FRCs, FDCs are found exclusively in the B cell follicle, where they support B cell homeostasis, maintain the follicular architecture, and promote robust humoral immune responses (13, 17–19). FDCs express complement receptors (CRs)-1 and -2 and can be induced to express Fc-gamma receptor (FcγR) IIb (17, 20, 21), which are important for their retention of immune complexes (ICs). FDCs also release ICs in the form of iccosomes (22), so that B cells can acquire Ag and present it to follicular helper T cells (Tfh). FDCs also provide costimulatory signals that enhance B cell proliferation and antibody (Ab) production (23).

An essential step for primary B cell responses is the germinal center (GC) reaction, which is a complex microenvironment that supports B cell clonal expansion and affinity maturation in response to T-cell-dependent Ags. GCs are critically influenced by the establishment of a functional FDC network capable not only of retaining Ag–Ab complexes through complement- and Fc-receptors but also of promoting the survival of GC B cells (24–26). FDCs are prominent in the light zone of GCs, where they facilitate B cell selection by displaying Ags (17, 21).

During the GC reaction, cognate interactions between Tfh cells and GC B cells are critical for the follicular T cells to provide the necessary signals for GC B cell survival and/or differentiation. CD40-ligand (CD40L) and IL-4 are among the crucial molecules of the T cell help to B cells and require close cell–cell interactions. It is established that Tfh cells are needed to maintain and to regulate GC B cell differentiation into Ab-secreting cells (ASCs) and memory B cells (27). ASCs and memory B cells provide both immediate as well as long-term protection against re-infections (28–30). Importantly, immunoglobulin (Ig) class switching (CSR) and somatic hypermutation (SHM) of Ig V regions both occur in the GC (23). These activities are dependent on the enzyme activation-induced cytidine deaminase (AID), which is a protein specifically expressed in GC B cells (31, 32). As a result, this enzyme is very important for successful Ab responses (33, 34) and can be used as a marker of T-dependent B cell activation.

Given the importance of GCs, stromal cell populations and the expression of AID in the generation of primary Ab responses, we examined these structures and the cell types, as well as AID and the Ab production in the context of immune responses in newborn mice upon early immunization at birth. We showed that mice on the day of birth have poorly organized LNs with few B cells or FDCs. However, we found that immunization at birth accelerated the accumulation of both B cells and Thy-1+ T cells inside follicles, and promoted FDC maturation and FRC organization in neonates. Nevertheless, the GC response was still delayed and reduced in neonates as compared to that in adults. Importantly, relatively few B cells in neonatal LNs expressed AID and as a result, they had fewer IgG-ASCs and lower IgG titers than adults did. Interestingly, the Ag-specific IgM response in neonates was similar to that in adults. These results suggest that despite an accelerated structural maturation of LNs in neonates following vaccination, the B cell response is still reduced in its ability to isotype switch.

## Animals and Methods

### Animals and Immunizations

Adult and female pregnant C57BL/6 mice were obtained from the animal facilities (UPEAL) of the Center for Advanced Research (CINVESTAV-IPN). This was arranged to ensure that by keeping the animals under discreet careful observation we could use the pups immediately at birth, which was considered day 0 in our studies. Mice were immunized with 100 µg of dinitrophenyl-conjugated keyhole limpet hemocyanin (DNP-KLH, Calbiochem), in combination with the adjuvant, Titermax Gold (Sigma, 1:10 v/v), *via* a single subcutaneous injection in the right inguinal region. Immunized pups were kept with mothers until weaning at 3 weeks. Serum was collected from anesthetized mice by cardiac puncture. Mice inoculated with sterile pyrogen-free saline solution (SS) were used as controls.

For the maternal transfer experiments, female C57BL/6 mice (6–8 weeks old) were immunized with DNP-KLH or SS and the day of immunization these female mice were mated. Fourteen days post-first-immunization, pregnant mice were boosted with another dose of DNP-KLH. Serum was collected from pups and mothers at various times after birth. Anesthetized mice were bled by cardiac puncture. To have enough sera, at least three litters of pups were used for each time point of the experimental design. All experiments were performed in accordance with the institutional guidelines for animal care and experimentation (UPEAL-CINVESTAV-IPN). The protocol and procedures employed were reviewed and approved by the UPEAL-CINVESTAV Ethics Review Committee.

### Antibodies and Lectins

Immunohistochemistry on frozen sections of draining LNs was performed using Abs against B220 (RA3-6B2), MFG-E8 (FDC-M1) and Thy-1 (G7) from Pharmingen, ER-TR7 against FRCs (ER-TR7) from Serotec, and AID (mAID-2) from eBioscience. Horseradish peroxidase (HRP)-conjugated goat anti-rat IgG (Thermo Scientific 31470), biotinylated anti-rat IgG (Vector BA-4001), and HRP-conjugated streptavidin (Pierce 21124) were used as secondary reagents. The lectin, peanut agglutinin (PNA) was obtained from Vector Labs.

### Immunohistochemistry

At necropsy, the inguinal draining LN (DLN) was excised from each animal, immediately frozen in Tissue-Tek OCT compound (Sakura) and cut in 3-µm cryosections. Serial cryostat sections were prepared from each specimen, fixed in pure cold acetone for 20 min, air dried, and stored at −20°C until staining, which usually occurred within 3 days. Sections were used to detect B cell follicles (B220), T cells (Thy-1) and then mature FDCs (FDC-M1), or FRCs (ER-TR7), PNA-positive GC B cells, and AID-positive cells (mAID-2) *in situ*. The specimens were obtained from at least three mice at each time point. For single staining, sections were incubated for 1 h with 9% H_2_O_2_ to block endogenous peroxidase at room temperature (RT). Before specific Ab staining, non-specific binding sites were blocked with Tris-buffered saline (TBS) 1× containing 1% human serum and 1% bovine serum albumin. For immunolabeling, we incubated the sections for 1 h with optimal concentrations of monoclonal Abs (mAb). After washing with TBS 1×, the sections were incubated with HRP-conjugated rabbit anti-rat IgG for 1 h at RT, washed and then stained with a solution of 3,3′-diaminobenzidine (DAB; Sigma) and H_2_O_2_ in TBS 1× to determine the peroxidase activity, and then washed with TBS 1×. For double immunolabeling, the slides were treated again with H_2_O_2_ to quench the specifically bound HRP and then peroxidase activity was visualized with SG substrate (Vector) which produces a blue-gray colored reaction. Results were recorded using Image-Pro PLUS software for the Olympus BX51 system microscope. Pictures were analyzed by ImageJ 1.47v (National Institutes of Health, USA).

### Flow Cytometry Analysis and Assessment of Antibody-Secreting Cells (ASC)

Draining lymph nodes were suspended in sterile phosphate-buffered saline and then pooled to have enough tissues and cells. Cell suspensions of DLNs from adults and from neonates were first pre-treated with purified rat anti-mouse CD16/CD32 mAb (2.4G2 BD Pharmingen Cat No. 553141) to block FcγRs II/III and then stained for 30 min on ice with the following fluorochrome-conjugated Abs: rat anti-mouse CD19-APC (1D3 BD Pharmingen Cat No. 550992), rat anti-mouse CD138-PE (281-2 BD Pharmingen Cat No. 553714), and rat anti-mouse Gr-1-PercP (RB6-8C5 BD Pharmingen Cat No. 552093). Cells were then washed, re-suspended in Cytofix/Cytoperm Kit (BD Pharmingen Cat No. 54714) on ice for 10 min, and washed with 1× Perm Wash Buffer 1:10 (BD™ Phosflow Cat No. 557885). Cells were then intracellular stained with goat anti-mouse IgG-FITC (Jackson Labs Cat No. 115-095-205). Cells were acquired on a CyAn™ ADP Analyzer. Data were analyzed with FlowJo software v7.6.5 (TreeStar).

### ELISA

The 96-well EIA/RIA Immunoplates (Costar 3590, Cambridge, MA, USA) were coated with 5 µg/ml of DNP-KLH at 4°C overnight in a moist chamber. After washing and blocking, serial dilutions of serum samples were incubated at 4°C overnight in a moist chamber. After further washes, HRP-conjugated goat anti-mouse IgG (Bio Rad 170-6516) was added for 2 h at 37°C. The reaction was visualized by the addition of 2,2′-Azino-bis(3-ethylbenzothiazoline-6-sulfonic acid) diammonium salt (ABTS) substrate (Sigma-Aldrich A1888). Absorbance was measured at 405 nm in a Sunrise Tecan microplate reader, software Magellan v.3.0 (Switzerland). Relative Ab titers were calculated after plotting the optical density of each well against the serum dilution and were derived from the linear portion of the resulting curves.

### Statistical Analysis

Values are expressed as median ± the interquartile range. Differences between mean values were analyzed for statistical significance using the GraphPad Prism 5 Software (La Jolla, CA, USA), using the Mann–Whitney *U* test. In the case of multiple comparisons, Kruskal–Wallis test with the Dunn’s multiple comparisons were used. *P* values less than 0.05 were considered statistically significant (*P < 0.05; **P < 0.01; ***P < 0.001; ****P < 0.0001).

## Results

### Immunization at Birth Accelerates the Maturation of B Cell Follicles and FDCs

**Figure 1:**
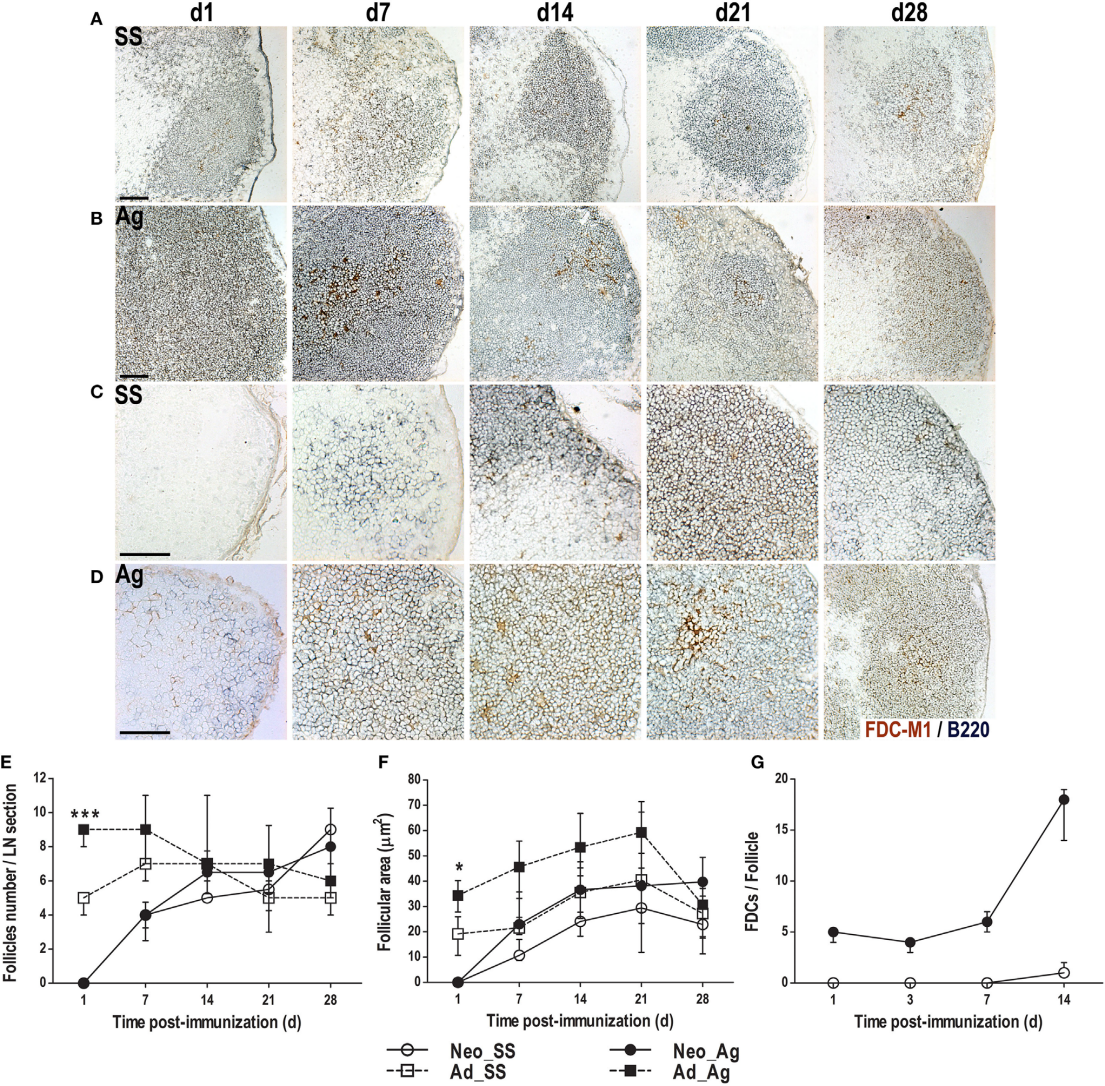
**Immunization at birth accelerates the formation of B cell follicles and appearance of follicular dendritic cell (FDC) networks**. Adult **(A,B)** or neonatal **(C,D)** mice were inoculated with sterile saline solution (SS) or DNP-KLH (Ag) and the draining lymph nodes were examined by immunohistochemistry on days 1, 7, 14, 21, and 28 after inoculating. Cryosections were probed with antibodies to FDCs (FDC-M1, brown) and B cells (B220, blue). The number of B cell follicles in each lymph node (LN) section **(E)** was counted and the area of B cell follicles in each section **(F)** was calculated. The number of FDCs **(G)** inside B cell follicles was determined in neonates during the first 14 days post-inoculation. Scale bar 100 µm. Data represent the median ± interquartile range from three to nine mice per group at each time point and were analyzed with Kruskal–Wallis test. Mice immunized as adults versus neonates immunized at birth. *P < 0.05, ***P < 0.001.

To determine the effect of immunization on LN architecture, we immunized neonatal mice (day of birth) and adults (6 weeks old) with either SS or DNP-KLH with Titermax Gold adjuvant and evaluated B cell follicles and FDCs by immunohistochemistry. As expected (35–37), we found well-formed B cell follicles and extensive FDC networks in adult mice, even before immunization (Figure 1A). Without immunization, the number of follicles per section remained steady (Figure 1E), and the follicular area expanded slightly over the next 4 weeks (Figures 1A,F). However, following immunization, the follicular area expanded dramatically especially in the first time points post-inoculation assessed (Figures 1B,F) and slightly more follicles were observed (Figure 1E). Nonetheless, both follicular size and number returned to baseline by day 28 after immunization (Figures 1E,F).

Regarding FDCs, while few of them were detected at different times in the control group (SS), Ag immunized adult animals showed a more prominent FDC mesh. Because in adult mice the FDCs were already forming dense networks inside B cell follicles, it was not possible to count individual cells in these animals. Accordingly, FDCs network was bigger at days 7, 14, and 21 in Ag immunized animals than in control groups (Figures 1A,B). By day 28, however, there were no apparent differences for both, the B cell follicles and the FDCs networks between Ag immunized and SS inoculated (control) adult mice. Thus, by day 28, the *in situ* cellular response generated to the Ag appeared to decrease to pre-immunization basal stages in adult mice.

In control LNs from neonatal mice, we observed very few scattered B cells on day 1, with no apparent follicular organization or FDCs (Figure 1C). We already found B cell follicles in LNs of control mice on day 7, but only minimal evidence of FDCs (Figure 1C). However, both B cell follicles and FDCs were clearly evident on days 14, 21, and 28, even without immunization. At these times, the number of follicles and their size resembled those of adult LNs (Figures 1E,F).

In contrast, immunization of newborns triggered the accumulation of B cells forming follicles-like below the SCS as early as 1 day later (Figure 1D). Likewise, FDCs were also observed at this time following immunization. In these animals, DLNs by day 7 exhibited the typical B cell follicles with FDCs inside, as seen in adult animals (Figures 1A,B). At this time, FDCs in immunized newborns were found in clusters and were more apparent than in control SS neonates. Thus, in immunized neonates, it was possible to quantify individual FDCs since day 1 until day 14 post-immunization (however, by day 21, FDCs were in a crowded cell mesh making very difficult to count them). The number of FDCs inside B cells follicles was bigger in immunized neonates than in the control animals during this period (Figure 1G). Bigger follicular areas and more prominent FDCs were maintained in the Ag-inoculated neonates in the following days (days 21 and 28) of the experimental protocol (Figures 1C–F). Thus, early experimental Ag exposure somehow accelerated the adult-like organization in neonates immunized at birth (day 0).

As shown in newborns, the quantity of follicles increased rapidly being very similar between the two groups (Ag-immunized and SS control, with an average of four follicles per section/node by day 7); reaching adult-like levels by day 14 (Figure 1E). In adults, the follicle number increased after 1 day post Ag immunization, declining to basal levels by day 21. Since in both the adults and the neonates experimentally immunized, the follicles were apparently bigger in size than in the control SS animals (Figures 1A–D); we measured the follicular areas at each time point in the different groups (Figure 1F). In adult mice, the follicular area was almost double in size 1 day after immunization than that of the SS control group. This ratio continued until day 21, declining after and being similar in both groups by day 28. In the newborns immunized at birth, the follicular areas at day 7 also had at least doubled the size of follicles from the control SS neonates. Bigger follicles in Ag immunized newborns continued by days 14 and 21. By day 28, the follicular areas were comparable between the two groups of newborns. It is worth mentioning that after day 7 post-immunization, follicular areas in neonates were rather similar to that of adults.

### Immunization at Birth Triggers the Rapid Reorganization of the FRC Network

**Figure 2:**
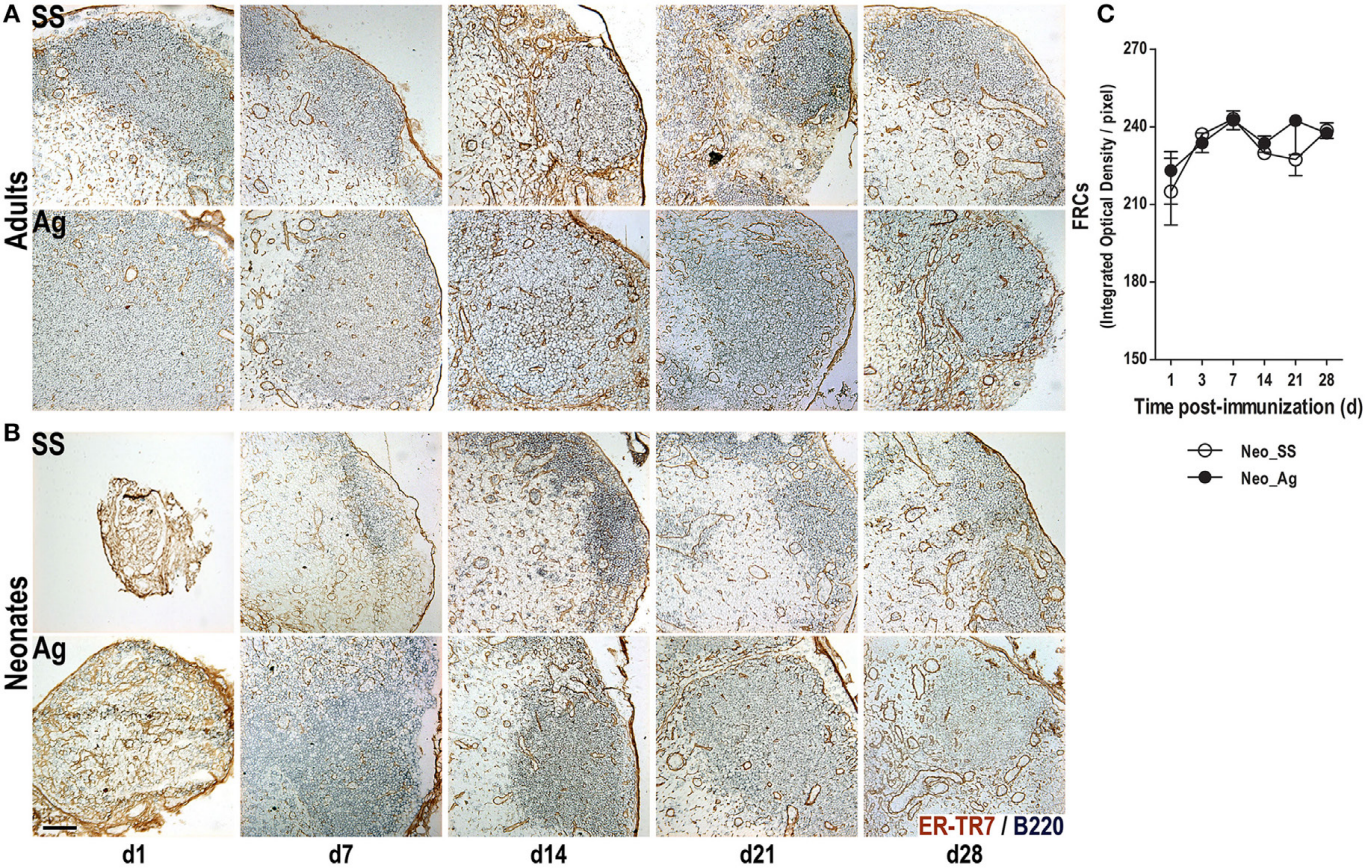
**Immunization at birth expands the fibroblastic reticular cell (FRC) networks**. Adult **(A)** or neonatal **(B)** mice were inoculated with sterile saline solution (SS) or DNP-KLH (Ag), and the draining lymph nodes were examined by immunohistochemistry on days 1, 7, 14, 21, and 28 after inoculation. Cryosections were probed with Abs for B lymphocytes (B220, blue) and FRCs (ER-TR7, brown). The integrated optical density of the DAB color (FRCs color quantity) by pixels was determined in control and in immunized neonates **(C)**. Scale bar, 100 µm. Data shown are pooled from three independent experiments and represent the median ± interquartile range.

Fibroblastic reticular cells are another important type of stromal cells needed for the efficient induction of adaptive immune responses (7, 8, 10, 13–15, 38, 39). To test whether FRC networks were expanded following immunization in adults and neonates, we probed tissue sections using the ER-TR7 Ab. As expected in LNs from control adult mice, we found FRCs in the T cell area and to a lesser extent in the B cell follicle and particularly at the T:B border surrounding the B cell follicles (Figure 2A). FRCs also delineated large blood vessels that were likely HEVs, both in control and in immunized mice. Nevertheless, this FRCs conduit system in adults appears better structured 1 week after experimental immunization (Figure 2A).

Interestingly, we observed robust staining for FRCs in neonatal LNs, even before immunization (Figure 2B). In fact, at day 1, the FRC network extended through the entire LN without interruption, probably because the B cell follicles are not yet formed. The FRCs network in the control neonates appeared (and was semi-organized) by day 7, whereas in immunized neonates, this network is clearly seen as early as 1 day after immunization. We tried to quantify the FRC networks in neonates by measuring the integrated optical density (IOD) of the brown color (produced by DAB) in neonates. Although the IOD between controls and Ag-immunized animals was rather similar, FRC networks were better structured in the Ag-inoculated neonates than in non-immunized ones (Figure 2C). Thus, Ag immunization at birth seemed to accelerate in the neonates the adult-like organization of FRCs too.

### Immunization at Birth Promotes the GC Reaction

**Figure 3:**
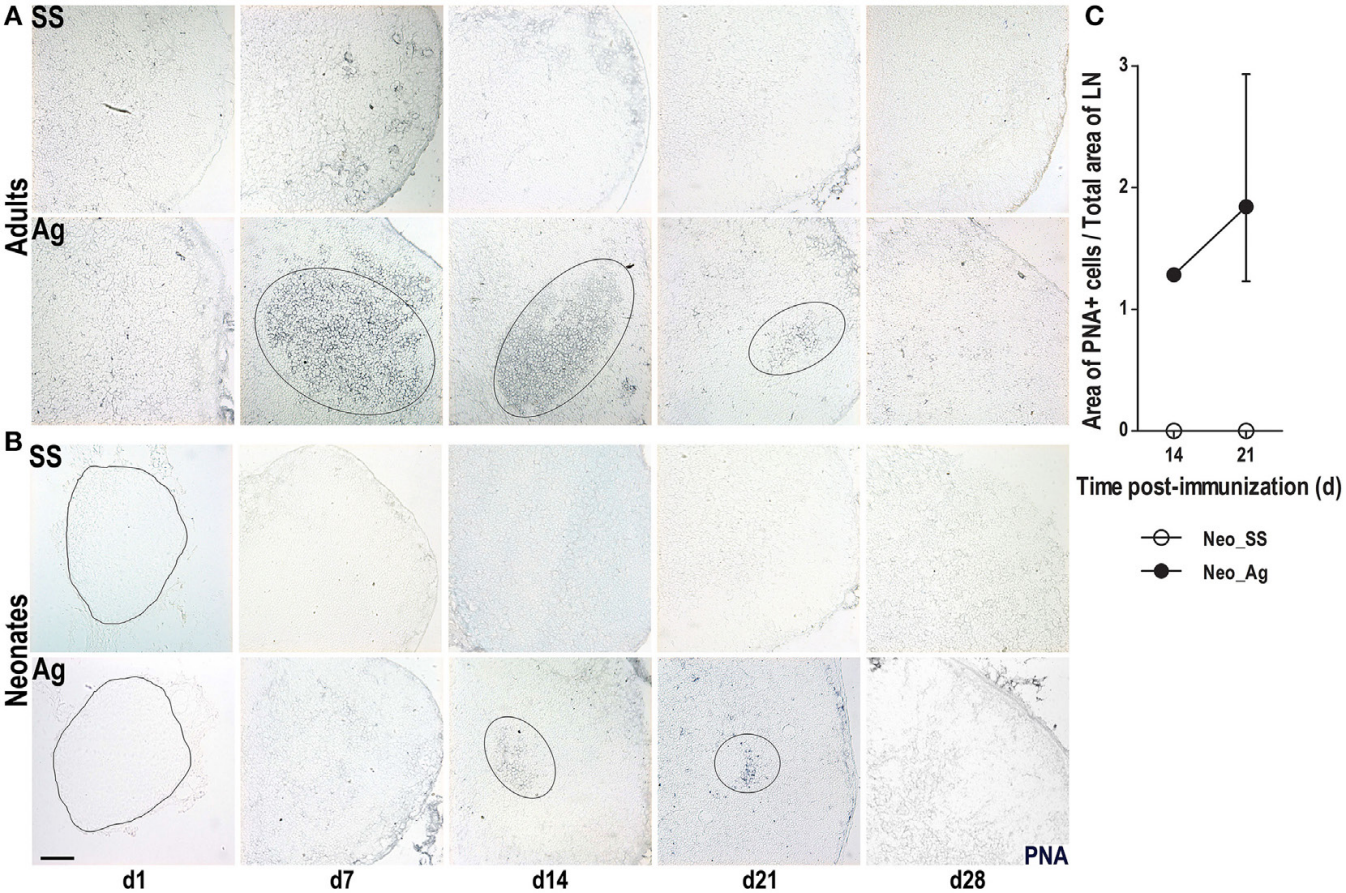
**Germinal center responses are delayed in neonatal lymph nodes (LNs)**. Adult **(A)** or neonatal **(B)** mice were inoculated with sterile saline solution (SS) or DNP-KLH (Ag) and the draining lymph nodes were examined by immunohistochemistry on days 1, 7, 14, 21, and 28 after inoculating. Cryosections were probed with the lectin peanut agglutinin (PNA, blue). Germinal centers (GCs) are delimited by elliptical lines. The outline of LNs at day 1 in neonates is indicated by dashed lines. **(C)** The GC area of control versus immunized neonates is represented as percentage of the Area of PNA+ cells from the total lymph node (LN) area on days 14 and 21. Scale bar, 100 µm. Data shown are pooled from three independent experiments and represent the median ± interquartile range.

The preceding data suggested that immunization at birth accelerated the architectural organization of LNs. To test whether the accelerated architectural maturation promotes a more efficient immune response, we assessed the capacity of B cells to undergo GC reactions. To accomplish this goal, we probed tissue sections with the lectin PNA. As expected, adult mice that received SS did not have PNA+ B cells at any time consistent with the absence of GCs in these mice (Figure 3A). However, immunized adult mice generated large PNA+ B cell clusters starting on day 7, a pattern that continued on day 14, and waned by days 21 and 28 (Figure 3A). Similarly, we did not observe any PNA+ B cell clusters in neonatal mice that received SS. However, following immunization, neonatal mice did develop small clusters of PNA+ B cells by day 14 (Figure 3B), which were still seen by day 21, but were difficult to detect by day 28. While PNA+ B cell clusters were absent in control SS neonates, they occupied about 2% from the total LN area in neonates at day 21 post-immunization (Figure 3C). These data suggest that neonatal mice are capable of generating a GC response following immunization at birth, but that GC formation is delayed and importantly attenuated compared to adults.

**Figure 4:**
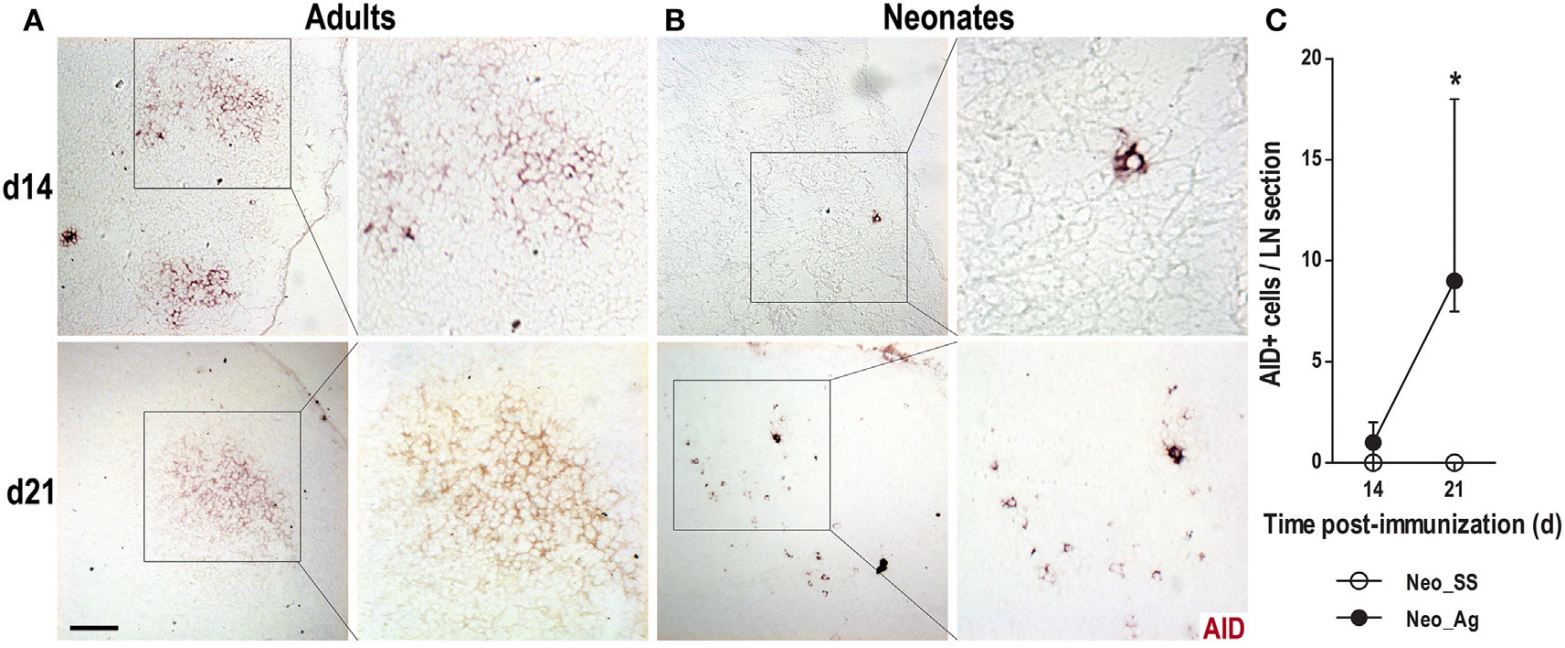
**Neonate lymph nodes (LNs) show few activation-induced cytidine deaminase (AID)+ cells after immunization**. Adult **(A)** or neonatal **(B)** mice were immunized with DNP-KLH (Ag), and the draining lymph nodes were examined by immunohistochemistry on days 14 and 21 after inoculating. Cryosections were probed with an antibody against AID (red). **(C)** The AID+ cells per LN cryosection in control and immunized neonates are shown on days 14 and 21. Insets show enlargements of indicated areas of AID+ cells. Scale bar, 100 µm. Data represent the median ± interquartile range from three to nine mice per group at each time point and were analyzed with Mann–Whitney *U* test. Control neonates versus neonates immunized at birth. *P < 0.05.

We next tested whether B cells expressed AID, the enzyme responsible for both CSR and SHM, two key molecular mechanisms that occur within GCs (33, 34). In immunized adults, we found well-defined clusters of AID+ cells on days 14 and 21 post-immunization (Figure 4A); however, AID+ cells were never observed in the control SS adult animals (not shown). In neonates immunized at birth, we observed AID+ cells at days 14 and 21 post-immunization (Figures 4B,C), although these cells were relatively rare and were observed scattered about rather than in clusters that resembled GCs. Again, we did not observe AID expression in newborns treated with SS (not shown). Altogether, these data suggest that newborns are capable of generating GC reactions, including AID expression, following immunization. However, this response seems to be delayed and very much reduced compared to that in adults.

### Immunization at Birth Prompts the Rapid Appearance of T Cells Inside B Cell Follicles

**Figure 5:**
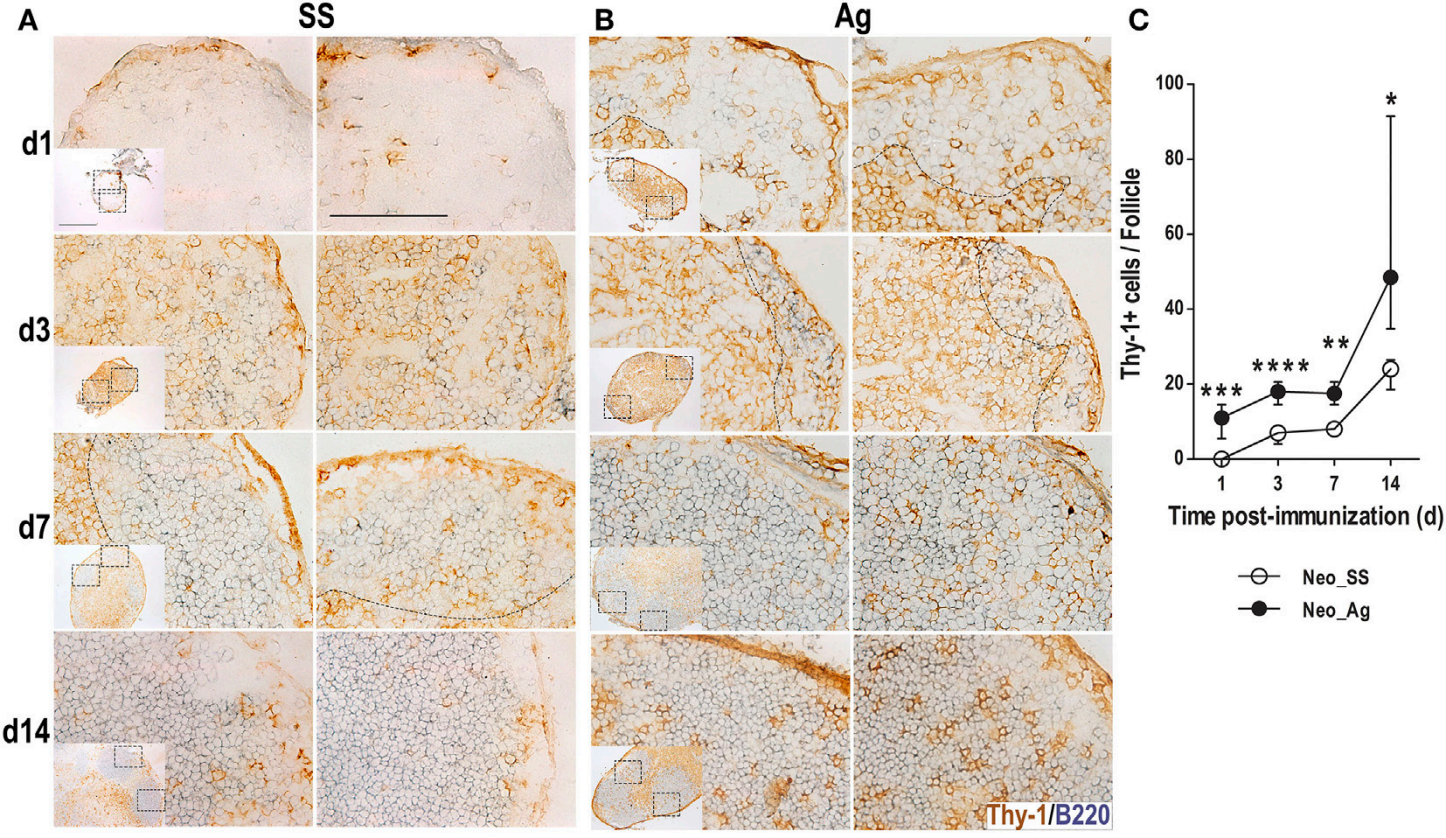
**Immunization at birth accelerates the recruitment of T cells inside B cell follicles**. Neonatal mice were inoculated with sterile [saline solution (SS) **(A)**] or DNP-KLH [Ag **(B)**], and the draining lymph nodes were examined by immunohistochemistry on days 1, 3, 7, and 14 after inoculation. Cryosections were probed with Abs for B lymphocytes (B220, blue) and T cells (Thy-1, brown). Two B cell follicles (scale bar 100 µm) are shown from each lymph node (insets, scale bar 300 µm). The number of (Thy-1+) T cells inside B cell follicles **(C)** from neonates was determined during the first 14 days post-inoculation. Data represent the median ± interquartile range and were analyzed with Mann–Whitney U test. Control neonates versus neonates immunized at birth. *P < 0.05; **P < 0.01; ***P < 0.001; ****P < 0.0001.

As GC reactions develop in response to T-cell-dependent Ags and because GCs were observed in neonates by day 14 post-immunization (14 days after birth), we searched for T cells inside follicles in the preceding days. A requisite for T cells to interact with follicular B cells is the recruitment of T cells into B cell follicles. Thus, we probed tissue sections with Thy-1 Ab to quantify the Thy-1+ T cells inside (B220+) B cell follicles at days 1, 3, 7, and 14 post-immunization. Similarly to B cells, in control LNs from neonatal mice, we observed very few scattered T cells on day 1, these T cells increased over time, some were inside B cell follicles (Figure 5A). By contrast, in immunized neonates, Thy-1+ cells inside B cell follicles were readily detected from 1 day post-immunization (Figure 5B). In the following days (3, 7, and 14) increasing numbers of these T cells inside B follicles were found in the Ag-inoculated neonates (Figures 5B,C). Thus, experimental Ag exposure at birth somehow accelerated the recruitment of T cells into B cell follicles.

### Immunization at Birth Elicits IgG-Secreting Cells and Ag-Specific IgGs

**Figure 6:**
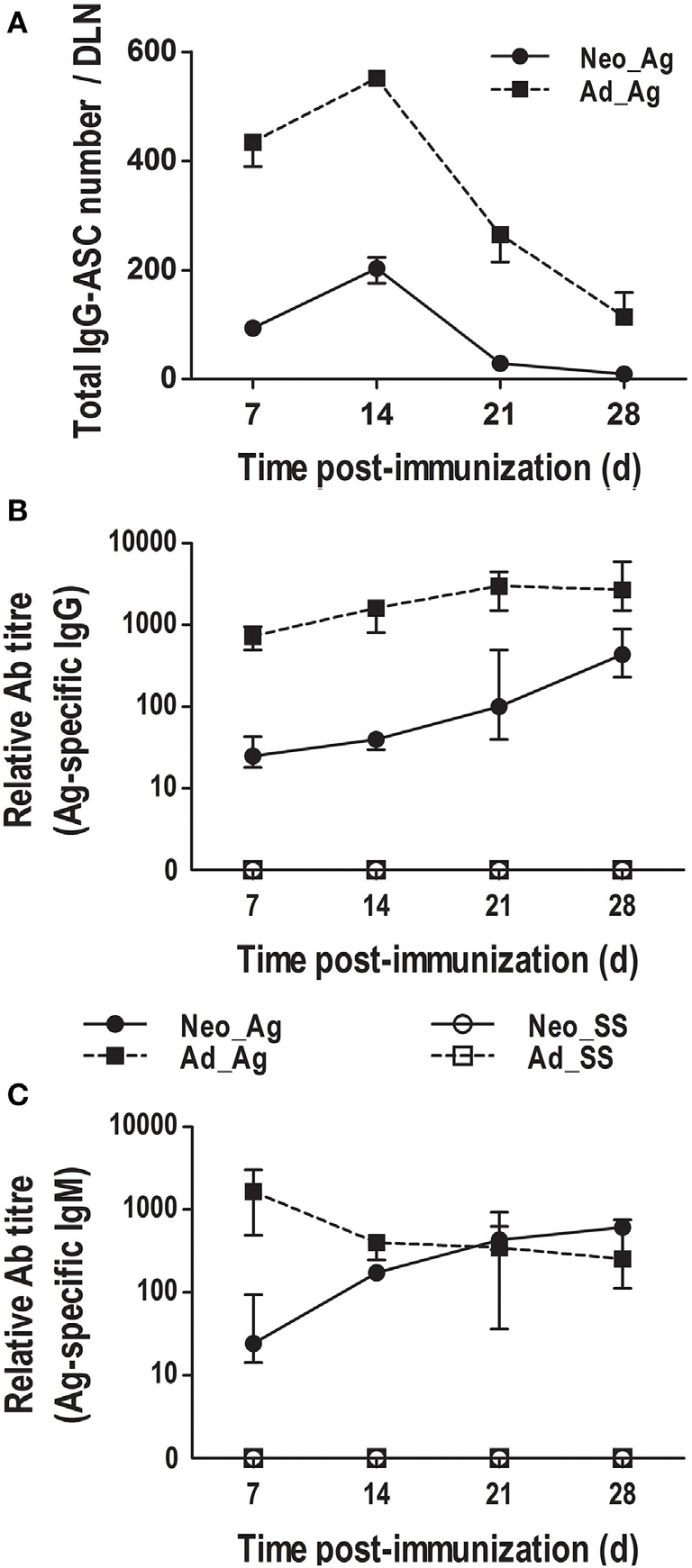
**Class-switched antibody (Ab) production in neonates immunized on the day of birth**. Adult or neonatal mice were inoculated with sterile saline (SS) or DNP-KLH (Ag). **(A)** The number of IgG-secreting cells (IgG-ASCs) was quantified on days 7, 14, 21, and 28 in draining lymph nodes (DLNs) using flow cytometry. **(B)** The titers of Ag-specific IgGs in serum were determined at days 7, 14, 21, and 28 by ELISA. **(C)** The titers of Ag-specific IgMs in serum were determined by ELISA. Data represent the median ± interquartile range from three independent experiments with three to nine mice per group at each time point.

As we found that neonates immunized at birth underwent GC reactions and also that T cells were present within follicles, we next wanted to know whether these neonates produce class-switched Abs. Therefore, using flow cytometry, we first enumerated total IgG-secreting cells in the LNs after immunization. We found that IgG-ASC numbers in immunized adults were already high by day 7, reached a peak by day 14, and declined by days 21 and 28 (Figure 6A). Interestingly, in neonates immunized at birth, IgG-ASC numbers also peaked at day 14 and fell by day 21 (Figure 6A). Although the IgG-secreting cell numbers in immunized neonates were much lower than those in adults, they followed a very similar kinetic to that of immunized adult mice.

We also measured Ag-specific serum IgGs in both adults and neonates at 7, 14, 21, and 28 days post-immunization (Figure 6B). In adult animals, we found that the titers of Ag-specific IgGs were already very high 1 week after immunization and increased steadily on days 14, 21, and 28. By contrast, in neonates immunized at birth (day 0), the titers of Ag-specific IgGs were low for the first 2 weeks, but they clearly increased by day 21 and were still increasing by day 28. Nevertheless, the titers of Ag-specific IgGs were generally about 10-fold higher in adults than in neonates, consistent with the size of GCs and numbers of AID-expressing B cells.

We also examined Ag-specific IgMs. As expected, we found that adults produced high titers of Ag-specific IgMs by day 7 post-immunization and that the titers declined by day 14 (Figure 6C). The kinetics of Ag-specific IgGs and IgMs in adults were as expected, whereas the circulating IgGs increased, the IgM Ab response decreased over time. However, in neonates immunized at birth, the kinetic of Ag-specific IgMs was similar to that of Ag-specific IgGs (Figures 6B,C). Of note, the Ag-specific IgM response in neonates was similar to that in adults.

### Ag-Naïve Pups Born to Immunized Mothers Have High Titers of Ag-Specific IgGs

**Figure 7:**
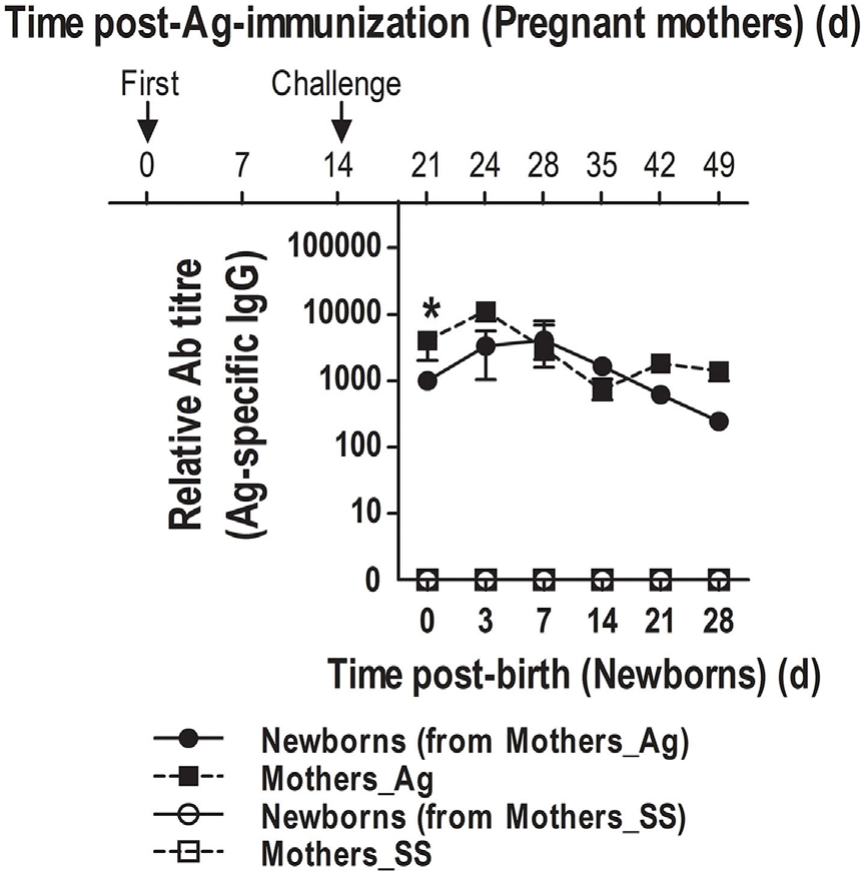
**Naïve neonates born to antigen (Ag)-immunized mothers show at birth Ag-specific IgGs**. Female mice were inoculated with sterile saline (SS) or DNP-KLH (Ag) and mated. Serum was obtained from the mothers and naïve pups at the indicated times and Ag-specific IgGs were measured by ELISA. Data represent the median ± interquartile range and were analyzed with Mann–Whitney *U* test. Immunized mothers versus naïve neonates born to immunized mothers. *P < 0.05.

Although neonatal mice were capable of generating class-switched IgGs and IgG-ASCs following immunization, this response was somewhat delayed compared to that in adults, suggesting that humoral immunity might be limited immediately after birth. Since it is known that maternal Abs could be passively transferred to neonates (40, 41), we investigated how newborns will cope with new foreign Ags to which the mother has been exposed in the surroundings, and most likely newborns will be too immediately after birth. Therefore, we tested whether at birth neonates will show Abs specific for the Ag that we have previously used to immunize the mothers. Thus, adult female mice were immunized with either SS or DNP-KLH and the day of immunization these female mice were mated. Once pregnant, mice were boosted with another dose of DNP-KLH 14 days after their first immunization and serum was obtained at various times after birth. We found that neonates born to immune mothers had high titers of Ag-specific serum IgGs on the day of birth. Titers of these Abs were maintained high during 3, 7, and 14 days after birth, peaking at day 7; however, the presence of these Ag-specific IgGs in non-immunized neonates declined by day 21 (Figure 7). Interestingly, the decrease of the passively transferred Abs by day 21 concurs with the time that neonates reach high titers of their own Ag-specific Abs (Figures 6B,C).

## Discussion

Early upon birth some tissues are still ongoing modifications to attain what is deemed the proper adult functioning. Although the immune system and SLOs are most likely no exception to this, the published research is contradictory (1, 2, 5, 42, 43). How the LNs in neonates are organized very early after birth and whether Ag plays a role in this process are not well known (16, 44).

We show in this work that immunization on the day of birth accelerated the FRC organization in neonatal LNs. FRCs, as part of the stromal cell supporting the LN architecture, organize the compartmentalization for B and T cells and direct the traffic of both, leukocytes and incoming Ags (13–15). Despite the importance of FRCs, there are very few publications about their organization in early postnatal stages (35). It has been shown that during the first week of life FRCs change dramatically and that by day 3, they are around the follicular area. During LN colonization with mature lymphocytes, B cells accumulate and generate follicles and the networks formed by the FRCs undergo progressive remodeling. These data are consistent with ours regarding “basal” conditions (i.e., in absence of experimental stimulus), as the distribution of FRCs 24 h after birth is shown throughout the LN, even in the subcapsular area, while B cells were sporadically observed. In the LNs from neonates immunized at birth and analyzed 24 h later, we already observed the accumulation of B cells in the subcapsular region, which were forming a sort of follicles. Interestingly, we found that the FRCs organization at day 7 in neonates that were immunized at birth is similar to that reported in immunized adults. It is neither clear whether B cells *per se* and/or the FDCs development are involved in this remodeling phenomenon nor which soluble and/or surface molecules might be taking part. Several concurrent mechanisms may lead to the progressive remodeling of this reticular conduit system (44). Progressive reorganization of FRCs networks from developing follicles may result from the loss of their integrity, facilitating the expansion of lymphoid organs and the restructuring reported in adults when facing antigenic or pro-inflammatory stimulation (10, 11).

On the other hand, despite the fact that FDCs represent the stromal cell compartment inside follicles, maintaining B cell homeostasis and organizing B cell responses in LNs (including the production of high affinity Abs), research on FDCs in early neonatal stages is very scant. Some reports described the absence of FDCs in 1-week-old neonates. However, when these mice were immunized and analyzed 1 week later (i.e., at 14 days of age), FDCs were then observed (35, 43). It thus seems that the presence of FDCs in very early neonates has been previously unrecognized. Certainly, we detected few FDCs as early as 24 h after experimental Ag immunization at birth, though not exactly within B cell clusters but rather at the side. This might not be unexpected since it has been reported that FDCs precursors are located immediately below the LN SCS, at the edge of follicles, and upon pro-inflammatory stimuli, they proliferate differentiating into FDCs (8, 45). In our protocol, the early antigenic challenge at birth might have provided the stimulation required to induce the appearance of FDCs (or FDCs precursors) at the edge of B cell follicles-like 24 h after Ag inoculation. In the non-immunized newborns, FDCs were observed until 14 days of life but not intrafollicular, just below the SCS where marginal reticular cells, their putative precursors, can be found. Thus, the behavior of these neonatal FDCs is consistent with the one described for FDCs in adults (17, 46). It is likely that this FDCs network might also provide positive co-signals for B-cell activation and differentiation at these early stages, but this requires much further research.

Regarding B cell development, at basal conditions in non-immunized neonates, we could see B cell follicles in the cortical area from day 7 after birth, which is consistent with previous reports (35, 47). This initial organization (even in the apparent absence of FDCs) could be due to the secretion of CXCL13 by the marginal reticular cells or by the presence of the lymphoid tissue inducers, with which the B cells establish a positive feedback for continuous secretion of CXCL13. CXCL13 has a dual role in the follicular compartmentalization of B cells: mediating B cell attraction and inducing increased LTα1β2 expression on the recruited cells. LTα1β2 then engages LTβRs on non-hematopoietic stromal cells, promoting differentiation/maturation of FDCs and leading to increased expression of CXCL13 to initiate the LN reorganization (48). Our data indicate that the antigenic stimulus prompts the functional reorganization of lymphoid tissues accelerating both the appearance and the structuring of cellular subcompartments as early as 24 h after stimulation.

Therefore, we decided to analyze the humoral response in the mice immunized at birth. Pihlgren et al. defined the neonatal humoral response as delayed, as they observed that ASCs reached their peak by day 14 whereas in adults peaked at day 10 after antigenic stimulus (43). Our work revealed that the humoral adaptive immune response (IgG) of the neonates immunized at birth was of a similar kinetic for ASCs than that of adults but of much lower magnitude. Moreover, Ag-specific IgGs were observed by day 7 in neonates immunized at birth, and PNA+ B cell clusters *in situ* were seen by day 14 post-Ag stimulation. Although PNA+ cell clusters have been described in mice immunized at 7 days of age and assessed 10 days later, i.e., at 17 days of age (49); we found these clusters at day 14 of age in neonates immunized just at birth. To this respect, the *in situ* GC response assessed by means of PNA labeling in neonates would be delayed (about 7 days) compared with that in adults. The presence of Ag-specific Abs by day 7, before the peak of the ASCs (day 14) could be because after BCR engagement, partially activated B cells can migrate to the T:B border and proliferate and differentiate there, creating clusters of proliferating B cells and ASCs (some even might undergo isotype switching). This is the extrafollicular (EF) response and those ASCs may last a few days as short-lived ASCs (50). On the other hand, some of these activated B cells might in turn migrate from the T:B border into follicles where they continue proliferating, forming a nascent GC. As a result, the long-lived ASCs from GCs maintain an increased Ab concentration. These data suggest that the Ab kinetic observed in the immunized neonates could be due first to an EF response, followed by GC reactions. Importantly, parallel to the decreasing of total ASCs within the DLNs from both adults and neonates, the systemic levels of Ag-specific IgGs were clearly augmented and were still increasing by day 28, revealing that the B cell response to Ags (including Ig class switching) can be mounted rather early after immunization at birth. Of note, this Ab response in neonates is also of lower magnitude than that in adults, but whether it is completely efficient needs further examination.

Since T cell help to B cells is critical for GC formation and for the generation of ASCs and memory B cells, and we found that neonates immunized at birth are able to induce the formation of GCs, we searched for T cells within the follicles. Although we did not perform a precise fine characterization of follicular T cells, our data show that the appearance of Thy-1+ T cells inside B cell follicles is accelerated upon very early immunization, compared to neonates in basal conditions. It has been reported that in early life the follicular T cells do not complete their differentiation into fully activated GC Tfh cells and thus fail to optimally support GC B cell differentiation and survival, compared to Tfh from adults. Whether this failure is due to the limited capacity of Tfh cells to localize in GCs is uncertain. In addition to this, the proportion of Tfh cells expressing Foxp3 seems to be significantly higher in neonates, suggesting a preferential induction of T follicular regulatory cells (49, 51). However, our *in vivo* data altogether suggest that these cells would be already operating at this early stage of life, revealed by the increasing numbers of T cells inside B cell follicles, the induction of GCs, and by the increase of ASCs and of Ag-specific IgGs over time.

A report of Ag-specific IgMs in neonates experimentally immunized 1 week after birth only mentioned (without showing the data) “the presence of certain quantities but minimal and transient IgMs” (42). However, we found that in neonates immunized at birth, Ag-specific IgMs and IgGs increased very similarly over time. In the case of adults, IgM decreased post-immunization concomitantly with the increase of IgGs, as previously characterized (52). In contrast to Barrios et al. (42), the differences with our work might be due to both the Ag and the time of inoculation we used. Regarding this, we wanted to test the capacity of the neonate immune system to an Ag to which they would be exposed precisely in similar quantities/conditions than adults, as it would be likely happening in the first days of extrauterine life. As B cell populations colonize peripheral SLOs in neonates, maybe a similar kinetic between the production of IgMs and IgGs might be expected. It suggests that B cells that generate IgM- and IgG-ASCs would be involved in the response at approximately the same time in these neonates. The regulation of IgMs and IgGs very early after birth has been poorly explored (53) and more studies are clearly needed. In adults, it is described that IgMs might act directly on B cells to regulate the magnitude of the IgG response (53–55). Due to the ability of IgM–Ag complexes to bind complement and activate the complement cascade, these might activate B cells by binding to CRs, providing important costimulatory signals. Another possibility is that Ag-free, pentameric IgM may engage Ag on Ag-bearing BCRs on B cells, triggering them (54). IgM would thus enhance the signals provided to B cells *via* the BCR (54, 56, 57). Something similar could be happening in neonates, enhancing the adaptive IgG response.

Furthermore, as the Ag-specific IgM response was similar between neonates and adults by day 14 post-immunization, it is conceivable that the EF reaction in neonates might be generating bigger quantities of IgM-ASCs compared to IgG-ASCs, as reported in adults (50). To this respect, the start of the IgG response would be delayed in neonates, whereas the IgM response would reach adult-like titers faster. Nonetheless, regarding Ag-trapping it is worth remembering that, as IgM is pentameric and IgG bivalent, it is conceivable that less quantities of IgM might compensate for the low IgG response during the first days after birth.

As mentioned, the enzyme AID acts in Ag-stimulated B cells allowing SHM and CSR. We found AID *in situ* by days 14 and 21 in the LNs of immunized neonates, AID+ cell clusters were fewer than in adults and seen in the cortical area where B cell follicles are present and where SHM and CSR could be happening. It has been shown in adult mice that during the immune response to a model Ag, specific B cells in LNs get initially activated at the follicle borders and differentiate into short-lived ASCs in EF foci (58, 59), which could be observed also in immunized newborns. Since AID-triggered CSR can also be induced during EF plasmablast differentiation (60), the presence of AID in newborns immunized at birth could represent the development of EF reactions. Besides the presence of GC reactions, the GC-independent CSR may represent an ancient pathway of AID induction that evolved before proper GCs appeared in evolution, as seen in lower vertebrates that develop ASCs and undergo CSR without GCs (60, 61). Of note, these neonates immunized at birth are capable of undergoing CSR since (a) we observed the presence of AID *in situ* after Ag stimulation and (b) we identified class-switched IgG Abs both by means of total IgG-ASCs in DLNs and of Ag-specific IgGs in circulation. Presumably, these neonates would be doing SHM too, but this needs to be demonstrated. Thus, compared to adults, the B cell response in neonates would be somewhat diminished in its ability to isotype switch, most likely due to poor AID expression. To the best of our knowledge, AID expression has not been previously shown in DLNs from neonates after Ag stimulation at birth.

As high titers of Ag-specific IgGs were not seen until day 21 post-immunization, we investigated whether neonates would be coping with totally new Ags immediately after birth through passively transferred maternal Abs, as it has been reported (40, 41). We found that the levels of Ag-specific Abs from both the mother and the naïve neonates were highly correlated. These passively transferred Abs confirmed a mother-derived coverage to neonates against Ags to which mothers, and most likely neonates, are exposed. This would ensure the protection of newborns while they structure the microarchitecture and subcompartmentalization within SLOs to efficiently mount their own responses.

Finally, besides the finding that rather early antigenic exposure (at birth) prompts the neonate immune system to engage in efficient immune responses, the type of Ag we used (62, 63) and the results we obtained are both indicative of efficient T cell help to B cells operating already at these early stages, although we did not examine this directly in this paper.

Thus, neonatal immune system might not be intrinsically immature but rather adapted for early postnatal life. Our results suggest that some responses of the neonatal immune system differ from immune responses observed in later life stages. However, it seems that evolutionary adaptations have provided these newborns with some elements to cope with Ags at birth.

## Author Contributions

Conceived and designed the experiments: RM-F, JY-P, and LF-R. Performed the experiments and the acquisition of data: RM-F, JY-P, EM-J, IG-H, and JC-A. Analyzed the data, interpretation, and discussion; wrote and revised the paper: RM-F, JY-P, AS-S, TR, and LF-R. Final approval of the version to be published: LF-R.

## Conflict of Interest Statement

The authors declare no financial or commercial conflicts of interest.

## Abbreviations

AID, activation-induced cytidine deaminase; ASC, antibody-secreting cell; CSR, class switch recombination; CR, complement receptor; DNP-KLH, dinitrophenyl hapten conjugated to keyhole limpet hemocyanin; DLN, draining lymph node; EF, extrafollicular; FcγR, Fc-gamma receptor; FRC, fibroblastic reticular cell; FDC, follicular dendritic cell; GC, germinal center; IC, immune complex; LT, lymphotoxin; PNA, peanut agglutinin; SLO, secondary lymphoid organ; SHM, somatic hypermutation; SCS, subcapsular sinus; Tfh, follicular helper T cells.
